# The effects of cool roofs on health, environmental, and economic outcomes in rural Africa: study protocol for a community-based cluster randomized controlled trial

**DOI:** 10.1186/s13063-023-07804-0

**Published:** 2024-01-16

**Authors:** Aditi Bunker, Guillaume Compoaré, Maquins Odhiambo Sewe, Jose Guillermo Cedeno Laurent, Pascal Zabré, Valentin Boudo, Windpanga Aristide Ouédraogo, Lucienne Ouermi, Susan T. Jackson, Nicholas Arisco, Govind Vijayakumar, Ferhat Baran Yildirim, Sandra Barteit, Martina Anna Maggioni, Alistair Woodward, Jonathan J. Buonocore, Mekdim Dereje Regassa, Tilman Brück, Ali Sié, Till Bärnighausen

**Affiliations:** 1 Heidelberg Institute of Global Health (HIGH), Faculty of Medicine and University Hospital, Heidelberg University, Heidelberg, Germany; 2 Centre de Recherche en Santé de Nouna (CRSN), Nouna, Burkina Faso; 3 Department of Public Health and Clinical Medicine, Sustainable Health Section, Umeå University, Umeå, Sweden; 4 Environmental Health and Occupational Health Sciences Institute, School of Public Health, Rutgers University, Rutgers, USA; 5 Department of Global Health and Population, Harvard T.H. Chan School of Public Health, Boston, USA; 6 Charité - Universitätsmedizin Berlin, Institute of Physiology, Center for Space Medicine and Extreme Environments, Berlin, Germany; 7 Department of Biomedical Sciences for Health, Università Degli Studi Di Milano, Milan, Italy; 8 Faculty of Medical and Health Sciences, University of Auckland, Auckland, New Zealand; 9 Department of Environmental Health, Boston University School of Public Health, Boston, USA; 10 Leibniz Institute of Vegetable and Ornamental Crops (IGZ), Großbeeren, Germany; 11 Thaer-Institute, Humboldt-University of Berlin, Berlin, Germany; 12 International Security and Development Center (ISDC), Berlin, Germany; 13 Africa Health Research Institute (AHRI), KwaZulu-Natal, South Africa

**Keywords:** Climate change adaptation, Heat exposure, Randomized controlled trial, Sub-Saharan Africa, Passive home cooling, Cool roofs

## Abstract

**Background:**

High ambient air temperatures in Africa pose significant health and behavioral challenges in populations with limited access to cooling adaptations. The built environment can exacerbate heat exposure, making passive home cooling adaptations a potential method for protecting occupants against indoor heat exposure.

**Methods:**

We are conducting a 2-year community-based stratified cluster randomized controlled trial (cRCT) implementing sunlight-reflecting roof coatings, known as “cool roofs,” as a climate change adaptation intervention for passive indoor home cooling. Our primary research objective is to investigate the effects of cool roofs on health, indoor climate, economic, and behavioral outcomes in rural Burkina Faso. This cRCT is nested in the Nouna Health and Demographic Surveillance System (HDSS), a population-based dynamic cohort study of all people living in a geographically contiguous area covering 59 villages, 14305 households and 28610 individuals. We recruited 1200 participants, one woman and one man, each in 600 households in 25 villages in the Nouna HDSS. We stratified our sample by (i) village and (ii) two prevalent roof types in this area of Burkina Faso: mud brick and tin. We randomized the same number of people (12) and homes (6) in each stratum 1:1 to receiving vs. not receiving the cool roof. We are collecting outcome data on one primary endpoint - heart rate, (a measure of heat stress) and 22 secondary outcomes encompassing indoor climate parameters, blood pressure, body temperature, heat-related outcomes, blood glucose, sleep, cognition, mental health, health facility utilization, economic and productivity outcomes, mosquito count, life satisfaction, gender-based violence, and food consumption. We followed all participants for 2 years, conducting monthly home visits to collect objective and subjective outcomes. Approximately 12% of participants (*n* = 152) used smartwatches to continuously measure endpoints including heart rate, sleep and activity.

**Discussion:**

Our study demonstrates the potential of large-scale cRCTs to evaluate novel climate change adaptation interventions and provide evidence supporting investments in heat resilience in sub-Saharan Africa. By conducting this research, we will contribute to better policies and interventions to help climate-vulnerable populations ward off the detrimental effects of extreme indoor heat on health.

**Trial registration:**

German Clinical Trials Register (DRKS) DRKS00023207. Registered on April 19, 2021.

**Supplementary Information:**

The online version contains supplementary material available at 10.1186/s13063-023-07804-0.

## Background and rationale

Ambient air temperature is rising due to anthropogenic climate change. Projections indicate that in a 4 °C world, monthly summer temperatures in sub-Saharan Africa will exceed 5 °C relative to the 1951 - 80 reference by 2100 [1]. Temperature increases and inadequate housing conditions in sub-Saharan Africa require adaptation to prevent excess heat exposure while indoors, especially in subsistence farming communities with limited to access home cooling technologies. Even in moderate temperatures, repeated heat exposure is associated with increased mortality and morbidity from non-communicable diseases (NCDs), including cardiovascular disease, heat stress, and heat stroke [2–8]. As life expectancy increases in sub-Saharan Africa, the disease burden from NCDs — exacerbated by increased heat exposure — is likely to rise [8]. Without interventions, rising air temperatures will lead to greater occupant exposure to extreme heat indoors, making passive home cooling a primary intervention pathway for supporting climate change adaptation [9] — particularly considering people are spending a greater proportion of time indoors [10].

Our current manuscript describes the aims and methods of a clustered randomized control trial (cRCT) on how sunlight-reflecting roof coatings, “cool roofs,” affect health, indoor climate, economic and behavioral outcomes in rural sub-Saharan Africa. This region currently suffers the largest disease burdens due to climate change worldwide [11, 12]. Our research question is whether cool roofs improve human health, indoor climate, economic, and behavioral outcomes. Our health endpoints include cardiovascular, metabolic, sleep, mental health, heat-related, and general health outcomes in home occupants of the Nouna Health and Demographic Surveillance System (HDSS) in Burkina Faso. We selected Nouna HDSS as our study community because it is located in sub-Saharan Africa and because demographic changes in Nouna, influenced by falling mortality rates, are likely to increase the size of heat-vulnerable groups (i.e., children and older adults) [8, 13]. Further, a resource-limited subsistence farming community, such as Nouna, is more significantly threatened by climate change and has substantially lower adaptive capacity than more well-resourced communities, making low-cost, passive interventions necessary to adapt to increased extreme heat.

### Temperature effects on non-communicable disease health outcomes

Studies in Africa have shown that moderate and extreme heat stress can have substantial adverse health and behavioral effects [7, 8]. High ambient temperatures have been associated with heat-related illnesses and increased cardiovascular mortality [14, 15]. Heatwaves (intense periods of high ambient temperatures) increase cardiovascular disease, heat stress, and heat stroke [2–6], as well as raise the risk of heat-related illness for individuals with diabetes [16]. Heat exposure has been shown to increase mortality and, to a lesser extent, morbidity from mental health outcomes in a global meta-analysis of 53 publications [17]. Increased heat exposure has also resulted in downstream reductions in worker productivity [18] and increased aggression [19]. In preliminary work, we found that moderate and extreme heat exposure in Nouna, Burkina Faso, increased premature death (years of life lost) from all non-communicable diseases [8]. A follow-on 16-year time series study in Nouna, Burkina Faso, revealed that extreme temperature increased the risk of death from climate-sensitive diseases, including cardiovascular diseases in the elderly [13].

### Existing evidence of housing interventions on health

Adaptive capacity is determined by access to resources, including cooling, in the context of housing [20]. The collective urban environment and individual housing mediate occupant heat exposure. For example, heat exposure in urban areas exhibit spatial variations, characterized by higher housing density, the prevalence of dark surfaces such as tar roofs and asphalt pavement, and sparse vegetation [21]. These urban “heat islands” are frequently found in economically disadvantaged communities [22]. Individual housing conditions can also mitigate the risk of infectious diseases, non-communicable diseases, and injuries due to altering abiotic (weather, hazardous construction, air pollution) and biotic (disease vectors, sanitation, crowding) exposures [23]. Interventions to promote home cooling will play an important role in facilitating adaptation in the face of climate change as global surface temperatures increase.

Previous studies have examined various interventions to mitigate indoor heat exposure, primarily focusing on higher-income urban settings. For instance, in the UK, the installation of external window shutters reduced heat-related fatalities by 30–60% during hot weather [24]. In urban European settings, implementation of passive cooling interventions on and around buildings, such as tree planting, cool roofs, and greenery facade, effectively lowered indoor temperatures, particularly in areas with limited air conditioning [25–28]. A global analysis revealed that access to air conditioning reduced the risk of dehydration and cardiovascular strain [29].

In contrast to urban, high-income settings, home occupants in low-resource rural settings in lower- and middle-income countries (LMICs) are particularly vulnerable to indoor heat exposure [30, 31]. Case studies sampling few households demonstrated that passive interventions can reduce indoor heat exposure in LMIC settings [32]. Cool roof technologies, such as solar reflective paints, Thermocol insulation, and ModRoof, have been shown to reduce daytime indoor air temperature by approximately 2.5 °C in both urban and rural areas of India [30, 33]. Housing design modifications incorporating screened windows and lightweight, permeable materials, while more resource-intensive, have also proven effective in reducing the indoor temperature by 2.3 °C in rural Tanzania [34]. Though evidence exists linking active and passive interventions to indoor heat exposure, few population-level pragmatic trials (using gold-standard randomized control trial or quasi-experimental methodology) have investigated the effectiveness of home cooling interventions on outcomes including individual health, environmental, behavioral, and economic outcomes — particularly in populations such as the Nouna HDSS.

### Housing features in Nouna and rationale for selection of the cool roof intervention

**Fig. 1:**
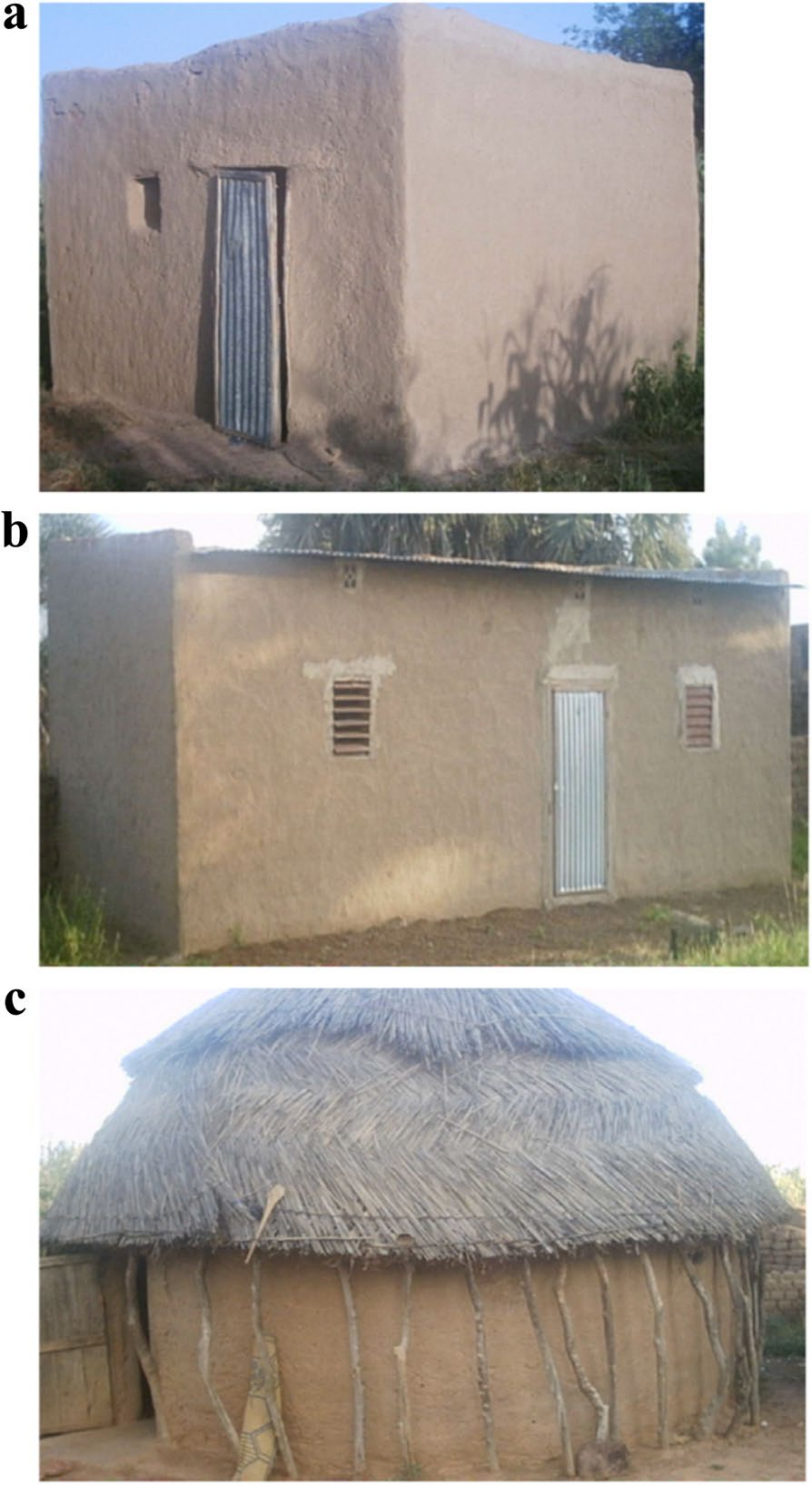
Prominent roof types in Nouna, Burkina Faso. **a** Traditional mud brick roof. **b** “modern” tin roof. **c** Thatch roof. Source: Schoeps et al. (2014)

We reviewed a range of passive home cooling interventions [35] to ascertain those most feasible and likely to be effective in rural Burkina Faso. Double glazing of windows, which reduces heat transfer between the in- and outside of a building, is likely less useful in rural Burkina Faso because houses have either no or only very small windows (Fig. 1). Wind catchers that deliver fresh air to home occupants require relatively large open spaces in housing, such as terraces or large windows, rendering this technology less applicable in rural Burkina Faso. Green roofs, which reduce indoor heat by absorbing sunlight, necessitate frequent rainfall or continuous irrigation for viability. Likewise, evaporative cooling systems utilize fountains that demand a significant water supply, which becomes scarce during Nouna's dry season. Thermal insulation may prevent solar heat from permeating the house but also prevent heat from leaving [36]. Additionally, in dwellings of solid wall construction, the application of insulation is quite expensive and challenging. We determined that cool roofs are the most feasible passive cooling technology for rural Burkina Faso based on their low cost, easy installation, direct application to existing housing stocks, and suitability to the Burkina Faso climate.

In a previous survey of housing conditions in Nouna, Burkina Faso, we found that average roof surfaces were 18 m^2^ [37]. Most homes in this area use mud bricks or “adobe” roofs coated with a fatty shea butter known as “beurre de Karité”, accounting for 75% of households in 2014 (Fig. 1a). Tin roofs are the second most prevalent, with 20% of house households using them (Fig. 1b), followed by thatch roofs at 4% (Fig. 1c). Tin roofs are increasingly prevalent with rising income and involve less maintenance than mud brick roofs. Both the traditional mud brick and the ‘modern’ tin roofs can be coated with a cool roof coating. Moreover, applying the cool roof coating to traditional mud brick-roofed homes effectively conceals the roofs, given that the roofs possess a slightly raised outer wall measuring 15 cm in height.

### Cool roofs — a detailed description

Cool roofs reduce indoor temperature by increasing solar reflectance (the ability to reflect visible wavelengths of sunlight, reducing heat transfer to the surface) and thermal emittance (the ability to radiate absorbed solar energy) [38]. In clear skies, roof surfaces absorb up to 95% of solar radiation [39]. Cool roofs can substantially reduce this fraction [40]. In addition, cool roofs have co-benefits of improving air quality and lowering carbon emissions [41, 42].

Liquid applied membranes (LAM), which are sprayed or painted onto the roof surface, are the most suitable cool roof technology for existing homes in Nouna, Burkina Faso because they can effectively coat surfaces with undulations or complex roofing structures such as mud bricks or corrugated tin and create a watertight and fire-retarding surface. Different LAM types have different Solar Reflectance Indices (SRIs), which indicate the level of solar reflectivity. LAM substances can also be easily transported and prepared onsite.

LAM-based cool roofs are compatible with a wide range of existing roof structures, incur no significant costs after installation, and are relatively cheap. Prices vary, however, depending on the location, manufacturing, and shipping of materials. Coating a low-slope roof, for example, can cost between $0.60 and $0.80 USD per square foot. The additional cost for cool roofs relative to conventional roof materials typically ranges between $0.00 and $0.20 USD per square foot for most roof materials [43].

### The knowledge gap — effectiveness of cool roofs in sub-Saharan Africa

**Fig. 2:**
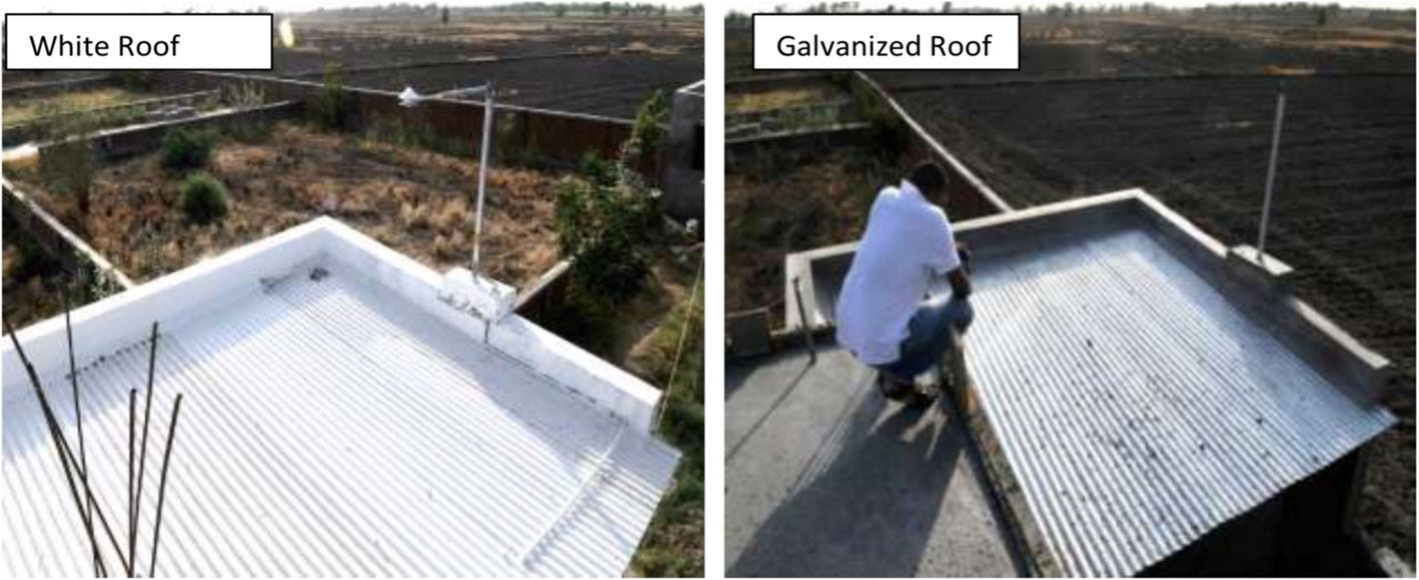
Galvanized cool roof vs. standard white roof in Gujarat, India. Source: Sahoo (2015)

Cool roofs have not been rigorously tested in sub-Saharan Africa, and previous studies were conducted in contexts with different climates and roof types. In Jamaica, applying cool roof coating on an uninsulated concrete roof (36 m^2^) led to a significant reduction of 4 °C in internal air temperature in April compared to the pre-coating measurements taken in March [44]. In Gujarat, India, a 9.3-m^2^ galvanized cool roof surface — Solar Reflectance Index (SRI) 0.9 — resulted in a 3.1 °C temperature reduction in comparison to a white roof of the same size and orientation in May (Fig. 2) [45]. Cool roofs installed on flat-roofed residential homes in the hot region of Andalusia, Spain, reduced overall electricity consumption by 2% and saved €59 million annually (compared to 2012 energy usage and prices), thereby preventing the emission of 136,000 tons of carbon dioxide (CO_2_) from electricity generation [46].

#### Aim

We aim to investigate whether retrofitting homes with a cool roof will affect the primary outcome — heart rate (as an indicator of physiological stress) — and secondary outcomes, including indoor climate parameters, blood pressure, body temperature, heat-related outcomes, blood glucose, sleep, cognition, mental health, health facility utilization, economic and productivity outcomes, mosquito count, life satisfaction, gender-based violence, and food consumption, among home occupants of the Nouna HDSS in Burkina Faso.

#### Trial design

**Fig. 3:**
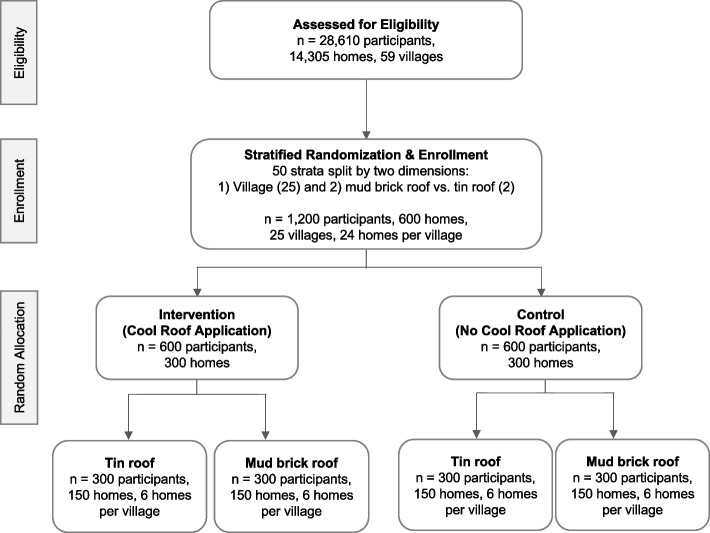
Trial design

This is a stratified cluster randomized controlled trial (cRCT) in Nouna, Burkina Faso (Fig. 3). The protocol was designed according to the Standard Protocol Items: Recommendations for Interventional Trials (SPIRIT) 2013 Statement [47]. The SPIRIT Checklist for this protocol is detailed in Supplementary Material, Additional File 1.

Our clusters are homes with roofs eligible for the cool roof intervention. Following the recommendation by Imbens et al. [48], we stratified our cRCT (rather than matching or using covariate information) using many small strata. We stratified on two factors: (i) village and (ii) the two types of roofing materials that exist in the study community (mud brick and tin). In each stratum, we randomized the same number of individuals (12, six women and six men) and homes (6) 1:1 to either receive or not receive the cool roof.

## Methods: participants, intervention, and outcomes

### Setting

**Fig. 4:**
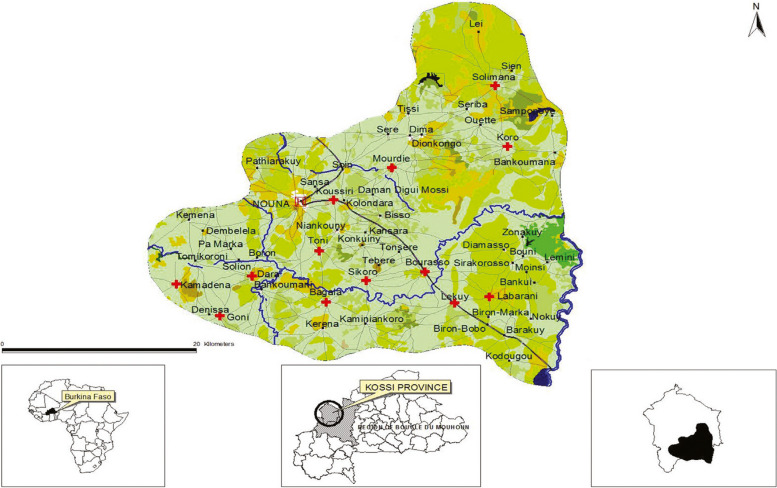
Map of the Nouna Health and Demographic Surveillance System (HDSS). Source: Diboulo, et al. (2012)

We are conducting this study in 600 clusters (homes) in Nouna, Burkina Faso. Nouna is a dry orchard savannah in sub-Saharan Africa characterized by a hot, dry summer (March to May) and heavy rainy season (June to September). Maximum temperature can reach above 40 °C, causing this subsistence farming community of 106,611 inhabitants (rural and semi-urban) considerable discomfort in summer months [49] (Table 1). The study location is an established HDSS community that has regularly collected population health data on vital life events (i.e., births, deaths, in/out-migration) since 1992 (Fig. 4).

**Table 1:** Temperature profile in Nouna, Burkina Faso

	**Minimum**	**25%**	**50%**	**Mean**	**75%**	**Maximum**
Temperature (°C)						
**Daily minimum**	3.3	21.1	22.8	23.1	25	32.8
**Daily average**	17.2	27.5	29.2	29.6	31.7	37.8
**Daily maximum**	22.8	33.3	36.4	36.1	38.9	43.9

### Trial participants (inclusion and exclusion criteria)

#### Eligibility for homes

Only homes located in the Nouna HDSS were eligible for this trial. For the safety of the research team, these homes are in areas that do not have security concerns. Conditions for exclusion include inaccessibility of the home, extensive damage to the roof, and the presence of roof materials other than mud brick or tin.

#### Eligibility for participants

All research participants must be at least 18 years old. We require that eligible participants reside in the Nouna HDSS and can consent to study participation. Incapacitated persons (i.e., immobile or unable to communicate) are excluded. Ethnicity, race, political orientation, religion, and class are not criteria for inclusion or exclusion in this study.

#### Intervention

In each village, we selected 12 houses with a mud brick roof and 12 houses with a tin roof. We randomized the houses in each category 1:1 to either receive the cool roof intervention or the control condition- no cool roof application.

#### Outcomes

**Fig. 5:**
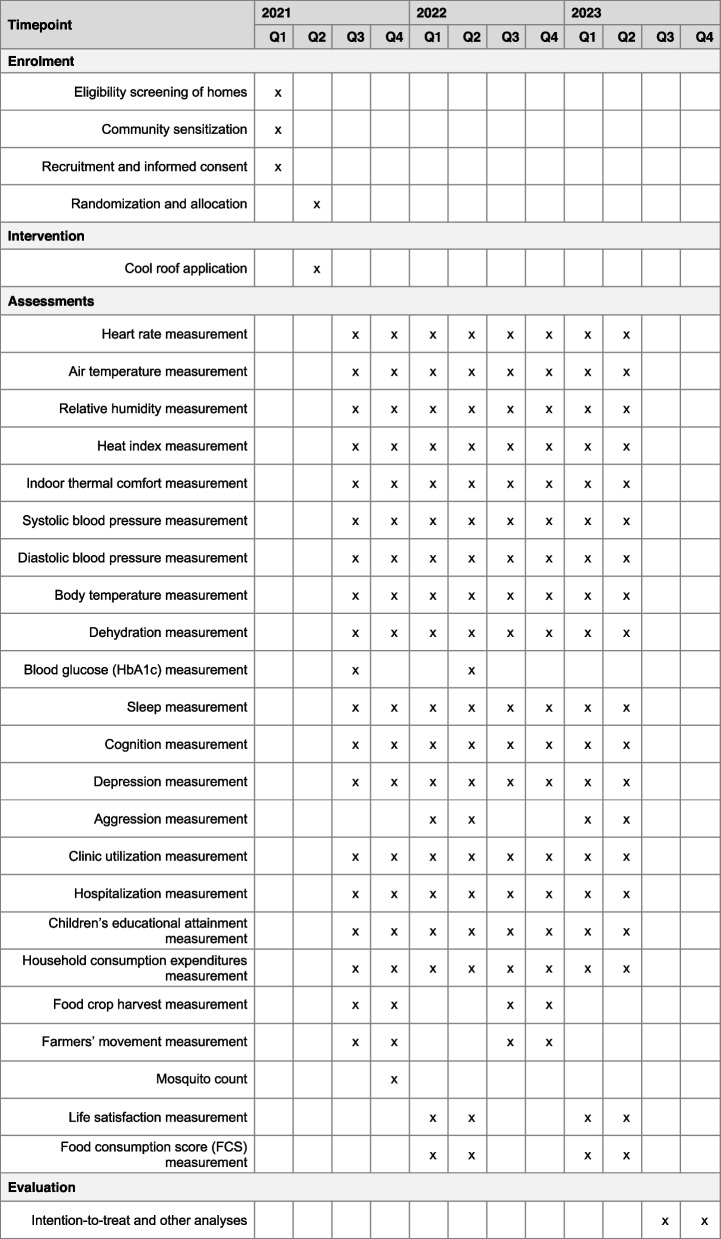
Schedule of enrolment, intervention, and assessments

Table 2 shows the primary and secondary endpoints, including the definition, functional form of the variable, and measurement approach. Figure 5 shows the schedule of enrollment, intervention, and assessment. The primary outcome is heart rate; we collect three measurements monthly from every participant during monthly home visits. In addition to the primary outcome, we are collecting data to assess the effects of cool roofs on (i) secondary environmental outcomes, including ambient temperature and humidity (measured using iButton sensors), and (ii) secondary health outcomes, such as blood pressure, body temperature, heat-related outcomes, blood glucose, sleep, cognition, mental health, health facility utilization, economic and productivity outcomes, mosquito count, life satisfaction, gender-based violence and food consumption. We randomly selected 152 participants [intervention, *n* = 76 (38 women, 38 men) versus control, *n* = 76 (38 women, 38 men)] to receive a smartwatch that continuously monitors heart rate, sleep, and activity. Between August 2021 and July 2022, our participants used the Withings Pulse HR smartwatch for data collection. In July 2022, owing to the fragility of the Withings Pulse HR, we replaced these devices with the Garmin Vivosmart 5 smartwatch. Several Withings Pulse HR smartwatches were breaking with participant use in the field, necessitating this replacement.

**Table 2:** Primary and secondary endpoints of the cRCT

**Primary endpoint**	
**Heart rate**	Definition: Heartbeats per minute Functional form: Continuous Measurement: Omron portable automated sphygmomanometers and smartwatch (in a subset of the cRCT population)
**Secondary endpoints**	
**Air temperature**	Definition: Ambient air temperature (in °C) Functional form: Continuous Measurement: DS1923 iButton Hygrochron heat and humidity measurement device
**Relative humidity**	Definition: Ratio of the amount of water vapor present in the air to the greatest amount possible at the same temperature (in %) Functional form: Continuous Measurement: DS1923 iButton Hygrochron heat and humidity measurement device
**Heat index**	Definition: Index that combines air temperature and relative humidity (using the formulae endorsed by the US National Oceanic and Atmospheric Administration (NOAA)) Functional form: Continuous Measurement: DS1923 iButton Hygrochron heat and humidity measurement device
**Indoor thermal comfort**	Definition: Current heat experience measured using heat strain score index. A score of < 13.5 indicates no or low heat strain, between 13.6–18 indicates potential for heat-induced illness, and > 18 indicates the onset of heat-induced illness Functional form: Categorical Measurement: Heat strain score index scale comprising 18 questions answered by individuals at the time of interview
**Systolic blood pressure**	Definition: Systolic blood pressure in mmHg Functional form: Continuous Measurement: Omron portable automated sphygmomanometers
**Diastolic blood pressure**	Definition: Diastolic blood pressure in mmHg Functional form: Continuous Measurement: Omron portable automated sphygmomanometers
**Body temperature**	Definition: Body temperature Functional form: Continuous Measurement: Inner ear thermometer
**Dehydration**	Definition: Loss of fluid greater than intake amount resulting in the body being unable to carry out its normal functions Functional form: Categorical Measurement: Urine hydration chart and question answered by individual
**Blood glucose (HbA1c)**	Definition: Average blood glucose measured over 3 months Functional form: Continuous Measurement: HemoCue® HbA1c 501 System to capture estimated average glucose (g/L)
**Sleep**	Definition: Number of hours slept and number of times the individual woke up Functional form: Count Measurement: Recall question answered by individual and smartwatch (in a subset of the cRCT population)
**Cognition**	Definition: The mental processes involved in gaining knowledge and comprehension Functional form: Continuous Measurement: Oxford Cognitive Screen (OCS)-Plus (assessing memory, language, visual-spatial ability, and executive functioning domains)
**Depression**	Definition: Self-reported depression. Each response category of “not at all,” “several days,” “more than half the days,” and “nearly every day” is assigned a score of 0, 1, 2, and 3, respectively. The total score is calculated by adding together the scores for the nine questions. The final scores of 0–4, 5–9, 10–14, 15–19, and 20–27 are the ranges for none, mild, moderate, moderately severe and severe, respectively Functional form: Categorical Measurement: Patient Health Questionnaire 9 (PHQ-9) to screen and monitor depression
**Aggression**	Definition: Self-reported aggression. Responses to 12 items rated on a 5-point Likert scale are summed up as a measure of aggression. Higher scores indicate higher aggressive behavior. The module can generate four subscales: physical aggression (items 1, 4, 8), verbal aggression (items 2, 5, 9), anger (items 6, 10, 12), and hostility (items 3, 7, 11). The score for each scale is the sum of the ratings for its items Functional form: Categorical Measurement: Short Buss-Perry Aggression Questionnaire (BPAQ)
**Clinic utilization**	Definition: Clinic utilization in the past 3 months Functional form: Count Measurement: Recall question answered by individual
**Hospitalization**	Definition: Hospitalization in the past 12 months Functional form: Count Measurement: Recall question answered by individual
**Children’s educational attainment**	Definition: Highest school grade completed Functional form: Count Measurement: Question answered by household proxy respondent
**Household consumption expenditures**	Definition: Amount (XOF) spent on monthly expenditures Functional form: Continuous Measurement: Locally validated set of questions covering a comprehensive spectrum of expenditure categories in rural Burkina Faso, answered by household proxy respondent
**Food crop harvest**	Definition: Harvest weight per field surface (yield) in tons/m^2^ Functional form: Count measurement Measurement: Remote sensing (Sentinel-2 satellite, validated against weighed crops in 2019)
**Farmers’ movement**	Definition: Work pattern of the farmers during their fieldwork Functional form: Continuous Measurement: Portable GPS device
** Mosquito count**	Definition: Number of mosquitos in the light trap (collected at 0600 h) and aspirator (collected at 0900 h) over defined night cycles Functional form: Count Measurement: CDC miniature light trap, aspirator
**Life satisfaction**	Definition: Overall life satisfaction level of an individual on a scale from 1 to 10 Functional form: categorical Measurement: Recall question answered by individual
**Food consumption score (FCS)**	Definition: Type and frequency of food intake over 7 days prior to the survey. The number of days a food item is consumed during the last 7 days before the survey is multiplied by a specific weight for the food group: starch staples (2), pulses (3), vegetables (1), fruits (1), fats (0.5), sugars (0.5), meat/fish/eggs (4), milk/dairy (4), and condiments (0). For each household, this leads to a score ranging from 0 to 112 Functional form: count Measurement: Recall question answered by female respondents

We are measuring the primary and secondary endpoints at varying times: every month during monthly home visits (including the primary endpoint, heart rate, and other individual health outcomes), continuously (home climate endpoints and smartwatch outcomes), and in two panel measurements over 1 year - the start of the trial and the end of the trial (long-term blood glucose — HbA1C).

Additionally, we are conducting three independent sub-studies using the cool roof trial infrastructure. First, we are quantifying the effect of cool roof use on mosquito abundance by counting mosquito abundance by species and gender in 120 households (60 with a cool roof versus 60 control homes) across ten villages at two weekly intervals over the rainy season. Second, we are quantifying the food crop harvest in each household’s fields and tracking household members’ work patterns using Global Positioning System (GPS) devices worn by the participants to compare farmer productivity in occupants of cool roof homes versus control homes. Third, we are quantifying the effect of cool roof use on food intake, psychosocial well-being, and gender-based violence.

### Timeline

Our trial began on April 20, 2021. The end of COVID-19 restrictions for home visits coincided with the beginning of the rainy season in June 2021. Due to this short window, we could not collect baseline information from participants. Therefore, we conducted an intensive campaign before the rainy season to apply cool roofs on 300 homes between April 23, 2021, and June 4, 2021. Our 30 cool roof applicators traveled across 25 villages, coating 54 homes per week. Each home required no more than 1 week to coat, including the curing time between preparing the roof surface, primer application, first and second coat application. We verified the iButton data loggers, which measure indoor temperature in each home, were collecting data.

After encountering challenges caused by the COVID-19 pandemic, we faced intermittent delays in three key areas during our research project. First, we encountered difficulties maintaining participant retention due to early equipment malfunctions requiring replacement. Second, due to the rapidly changing on-the-ground security concerns, we had to modify participant questionnaires and make the corresponding ethical amendments. Third, ongoing security concerns in Burkina Faso, including the military’s restrictions on fieldworker movement and data collection, posed additional obstacles to our research progress.

We obtained funding for our cRCT from the German Research Foundation (Deutsch Forschungsgemeinschaft, DFG) to execute the study in Nouna, Burkina Faso. Soon after securing funding, the security situation in Burkina Faso deteriorated, particularly in our study region. The primary security concerns included fear of attacks on villagers, including our participants, from Islamic insurgency groups. From this point onwards, we constantly interacted with our Burkinabe government partners (the National Institute of Public Health) and our community partners (CSRN and the community advisory board) to consider the advantages and disadvantages of implementing and continuing the study. We prepared contingency plans to relocate our cRCT to a more secure location, including to another country — Kenya. In the event of relocation, we did not want to publish a trial protocol that inaccurately stated the country or community of a cRCT because future reviewers of our trial publications would find it confusing and incorrect that we had published a protocol in Nouna Burkina Faso, but were attempting to publish the results of a potentially relocated study in Kenya. Through our ongoing safety and security monitoring process, we ensured participant safety and successfully executed our cRCT in the originally intended location of Nouna, Burkina Faso. For this reason, we are submitting this study protocol post-participant recruitment but before the last data collection on June 30, 2023.

### Sample size

Of the 59 villages in the Nouna HDSS, 25 were randomly selected (or about half of all the villages in the Nouna HDSS area), generating a total of 50 strata. We randomly selected 12 homes in each village with mud brick roofs and 12 with tin roofs. We randomized these homes (*n* = 600) in each category (1:1) to either the intervention (cool roof application) or the control condition (no cool roof application). The distribution of these 600 households across the trial groups was evenly balanced between mud brick and tin roof houses and between the intervention and control groups. Each of the 600 households enrolled in the study included one female and one male participant (*n* = 1200 participants).

### Power calculations

Prior non-randomized studies indicate that reducing the indoor temperature by 3–4 °C should decrease heart rate by several beats per minute [32, 50, 51]. Because of high uncertainty about the previous findings, their attributability to the climate and context in rural Burkina Faso, and the relatively large number of secondary endpoints, our cRCT is powered conservatively (Table 3). In the baseline power calculation, the minimal detectable heart rate difference between the cool roof intervention and control condition arm is 0.22 beats per minute (Table 3) for 300 homes (in the intent to treat) in both arms, assuming a significance level of 5% and power of 80%. We further assumed that 15% of homes refuse to participate in the trial and 15% of individuals would be lost to follow-up. Our sensitivity analyses showed that these power calculation results are highly robust to changes in intra-cluster correlation, the number of individuals in which the outcomes were measured per home, and the base correlation generated by our stratification (Table 3).

**Table 3:** Minimal detectable difference in heart rate — baseline power calculation and sensitivity analyses

No. individuals/baseline correlation	1	2	3
ICC = 0.1			
0	0.28	0.21	0.18
0.1	0.28	0.21	0.18
0.2	0.28	0.20	0.17
ICC = 0.2			
0	0.28	**0.22**	0.19
0.1	0.28	0.22	0.19
0.2	0.28	0.21	0.19
ICC = 0.3			
0	0.28	0.23	0.21
0.1	0.28	0.23	0.20
0.2	0.28	0.22	0.20

The significance level is 0.05

The table shows the minimal detectable differences in heart rate (in beats per minute) for 300 homes in each arm for a range of combinations of values of the intracluster correlation coefficient (ICC), the number of individuals per home, and the baseline correlation generated by our stratification

## Methods: assignment of intervention

We stratified households to cool roof intervention or control — first by village and subsequently by roof type (mud brick or tin). After identifying and assigning participants to village and roof type strata, we used a computer algorithm generated by the field team in Nouna to randomize and assign participants to the cool roof intervention or control arm. Therefore, we were able to balance the control and treatment arms by village and roof type covariates. Allocation concealment was ensured through only the site lead in Nouna having encrypted/password-protected access to the household data and the subsequent use of an automated computer-generated algorithm for randomization. Prior to randomization, there was no apriori knowledge of group assignments. The computer-generated allocation sequence used for randomization was not released until households consented to participate in the trial. The site allocation was revealed on March 31, 2021. This is a single-blinded study, where the households are aware of the intervention assignment and all researchers, except for two senior biostatisticians, are unaware of the household group assignments.

## Methods: data collection, management, and analysis

### Data collection plan

We are collecting trial data from a variety of sources for measuring health, indoor climate, economic, and behavioral outcomes (Table 2). Our primary outcome — heart rate — is collected monthly by trained field staff using an Omron portable automated sphygmomanometer. We present a complete list of secondary outcomes, including the associated instruments used for data collection in Table 2. Additionally, we are collecting continuous health indicators, including heart rate, sleep, and activity using a Withings Pulse HR or Garmin Vivosmart 5 smartwatch worn by a subset of participants.

We are collecting HbA1C blood samples - an indicator of long-term glucose control as two-panel measurements in the hottest months of 2021 and 2022, respectively, using the HemoCue® HbA1c 501 System. A qualified phlebotomist took 4 ml of whole blood by venipuncture for each participant. The phlebotomist labeled the collection tube with the participant's first and last name, and their household, individual and study identifying codes. Blood samples collected in different villages were stored in a refrigerator, and transfered to the laboratory within 48 h. Our laboratory technicians verified that HbA1c samples matched participants and recorded measurements systematically on examination forms according to protocols supplied by HemoCue®. Blood samples were subsequently disposed of in accordance with the laboratory's biological waste disposal procedure. We removed all identifiable information, except the individual identification code, prior to conducting data analysis.

We are collecting indoor temperature and humidity data using the DS1923-F5 iButton hygrochron temperature and humidity sensors. We are also conducting three independent sub-studies using the cool roof trial infrastructure that will be reported separately. First, we will investigate the effects of cool roofs on mosquito abundance, where light traps and aspirators are deployed during the rainy season to collect mosquito samples from a subset of households. Second, we will investigate the effects of cool roofs on farmer productivity, where GPS devices will record individual movement and remote sensing will provide data on crop harvest yield. Finally, we will investigate the effects of cool roofs on food consumption, psychosocial well-being, and gender-based violence from data collected during monthly home visits.

We are creating a secure SQL relational database hosted at Heidelberg University to unite individual data streams. We will merge data through unique identifying codes for each participant and subsequently clean data to remove duplicates and identify missing and inaccurate values. We will move cleaned data into the master relational database and record data management processes in a logbook. We are cleaning and consolidating data as it streams in from multiple sources.

We will validate objective measurements such as temperature and humidity against self-reported subjective measures such as heat stress index to gauge the thermal comfort of our study participants in control and intervention homes. Table 2 presents the full range of objective and subjective data collected.

### Data management

We are storing data using numeric identifier codes to maintain participant anonymity in a password-protected database that is only used for analysis by the research team. Access to this database is restricted to a minimum number of people, including the principal investigator (PI), co-investigators, biostatisticians, and data scientists. We follow the principles of long-term preservation and open access, described in the 2010 Principles for the Handling of Research Data adopted by the Alliance of German Science Organizations [52]. While no third party is privy to the original data (primary and secondary endpoints, as well as exposure status information), anonymized data will be made available through the website of the institution, which the PI leads [Heidelberg Institute of Global Health (HIGH), Faculty of Medicine and University Hospital, University of Heidelberg] 1 year after study completion. Restrictions to this open-access model will only apply if standard approaches to guarantee anonymity (removal of names and other personal identifiers) cannot guarantee anonymity (i.e. if triangulations of data from several data sources can reveal identities).

While the data will be openly accessible as described above, users must register (on the HIGH website) with a title and a short description of their research projects. Any publications resulting from analyses of the open-access data from this project will acknowledge funding through the German Research Foundation (DFG).

### Statistical analysis

#### Analysis of monthly home visit data

The analysis for our primary outcome, heart rate, will be performed on the principle of intention-to-treat (ITT). We will use generalized linear mixed models (GLMM) to analyze the causal effect of cool roofs on our primary and secondary endpoints. In addition to the base-case estimations, without covariate controls, we will also estimate the intervention effect of cool roofs by adjusting for covariates to boost statistical efficiency. We will adjust for clustering of standard errors at the household level.

To estimate the intervention effect of cool roofs on heart rate, we will report the effect estimates comparing intervention and control arms based on the hypothesis that effects will be greatest during the hottest month of the year (April). We will additionally report effect estimates at monthly intervals of data collection, except for HbA1c (which is measured in two time points), because we hypothesize the intervention effect will vary based on outdoor weather conditions during the trial period; therefore, we will include an interaction term between the intervention and the month of trial. We will perform additional sub-analysis by selected variables where relevant (i.e., by season, gender, age group, time of the day (day/night), and roof type). We will explore the use of different imputation methods for missing values in the covariates. We will conduct sensitivity analysis, including repeating analyses accounting for individuals lost to follow-up. All data analyses will be performed using R software.

#### Analysis of smartwatch data

Raw indoor and outdoor temperature and relative humidity data, collected in 15-min intervals, will be averaged at the hourly level. We will categorize environmental data as anomalies when measurements are two standard deviations beyond the corresponding monthly average values based on roof type (mud brick or tin roof).

We will analyze data from smartwatches on heart rate and sleep. First, for heart rate data, we will exclude duplicate heart rate measurements or those that exceed the age-predicted maximum heart rate as implausible values. We will round and aggregate remaining heart rate measurements to the nearest 1-h interval to align with summarized indoor and outdoor temperature and relative humidity data. To distinguish daytime and nighttime heart rates, we will use median sleep onset and offset to define nighttime from 22:00 to 06:00 h. Measurements will be analyzed if they reach the threshold of at least 1 h during the same day or night, with a minimum of one measurement every 15 min. The 15-min threshold was selected based on previous research [53]. Second, for sleep data, we will calculate sleep duration based on onset and offset time to eliminate errors and minimize outliers. Only overnight sleep periods, where nighttime sleep is defined as having a sleep onset of ≥ 17:00 and a sleep offset of ≤ 13:00, with a 2-h adjustment made to account for earlier bedtimes of the study population, will be included in the analysis. If a participant has multiple sleep observations in a single night, we will aggregate these into one sleep observation by adding the sleep duration values and the duration between the first offset and last onset to the wake time after sleep onset. We will exclude data where multiple sleep observations overlap for a participant. Furthermore, sleep detections with a time difference of fewer than 3 h between sleep onset and sleep offset will be excluded, in line with the manufacturer’s declaration that such detections represent invalid measurements.

We will evaluate the intervention effects of temperature exposure on heart rate and sleep outcomes using generalized additive mixed models, with individuals nested within households as random terms. Natural cubic splines will be used to evaluate non-linear effects. In cases where non-linear effects are not detected, linear terms will be substituted in the final models. We will present effect estimates of daily exposure averages on daytime versus overnight periods when individuals are most likely present in their homes. Furthermore, we will present intervention effects during the hottest month (April), monthly aggregates, and an overall aggregate for the entire study period. Where relevant, models with continuous daily or intraday time series data will include the day of the year and month indicators to account for time trends and seasonality, respectively. We will present supplementary models with an interaction term between the intervention group and roof type to assess variations resulting from the thermal characteristics of household vernacular architecture.

## Methods: monitoring

We have appointed an independent steering committee comprising three members: a climate change and health domain expert, a clinical trials expert, and a biostatistician to maintain project oversight throughout the study. The steering committee will convene three meetings, the last of which will be in June 2023. At these meetings, the research team will review the conduct of the trial, including a review of adherence to intervention components, logistical issues, and completeness and quality of collected data. Our biostatisticians will also present emerging effect estimates in the intervention and control arms to the steering committee, who will ascertain whether the results display:

1. Null finding(s) > indicating the study should stop
2. Significant finding(s) > indicating the study should stop
3. Emerging positive finding(s), but statistically insignificant > indicating the study should potentially continue, and therefore, the data collection should continue for a recommended duration.

The cool roof is a passive home cooling intervention and, therefore, required no human interaction or behavior change on behalf of our participants. We conducted an education program through our field staff to ensure study participants did not place items on their roofs that could obstruct the optimal performance of the cool roof. Our field teams regularly monitored roofs during monthly home visits and highlighted any deterioration or other issues for resolution. Throughout the trial, our study team exchanged regular communication via virtual and phone calls, emails, and short messages about trial progress.

We encountered, however, adherence issues with participants removing smartwatches, particularly at night. Our field team explained the importance of smartwatch data and encouraged non-compliant individuals to wear the device during home visits.

## Ethics and dissemination

### Research ethics approval

Our study protocol was approved by the Ethics Committee of the Medical Faculty of the University of Heidelberg on May 6, 2019 (S-293/2019) prior to the trial commencing. We registered the trial and study design with all primary and secondary endpoints and analytical approaches as trial DRKS00023207 in the German Clinical Trials Register (DRKS) (https://www.drks.de/drks_web/).

Our co-investigators are formally trained in research ethics through the US Collaborative Institutional Training Initiative (CITI). The co-investigators trained the data collection teams on research ethics. We guided our data collection team on facets of research ethics including principles of respect for persons (including autonomy, which includes the right for individuals to decline or withdraw from our trial, and the protection of vulnerable subjects), beneficence (the importance that we do no harm), and justice (in terms of the distribution of information and the inclusion of participants).

### Plans for communicating important protocol amendments to relevant parties

The Ethics Commission of the Medical Faculty of the University of Heidelberg was contacted and notified about any protocol amendments requiring their acceptance. Following the approval of the Ethics Committee and before implementation, all research members were informed about protocol amendments.

### Consent and withdrawal

Our trial will adhere to the principles outlined in the Declaration of Helsinki and the Belmont Report, which prioritize respect for persons, beneficence, and justice. We provided written study information and the informed consent form to eligible individuals. The study manager explained the study's aims and detailed procedures — in the presence of a witness if required. We provided sufficient time for our participants given to the subject to decide whether or not to participate in the study. Participants were given the opportunity to enquire about details of the study and any questions regarding the study, and responses were provided. For illiterate participants, we obtained a thumbprint signing and a witness’ signature to document consent before enrolment. We requested consent to collect blood samples to analyze the blood samples and any biological material. We obtained written, informed consent to participate from all participants.

Our trial participants were informed that consent can be withdrawn at any point in time, including the destruction of biological samples and deletion of collected data, without bearing any negative effects in terms of health care or otherwise. We will inform our trial participants about abnormal results emerging from data collection and refer them to the health center for further investigation and treatment. Enrolment of participants in the study will, therefore, ensure adequate treatment is initiated if there are any indications. Participants in our trial will not have to relinquish any concomitant care. We anticipate no direct harm as cool roof products applied to roofs have met regulatory safety standards and are not in direct contact with participants. We will repair any damage to the cool roof coatings during the course of the study.

We informed eligible households about the risks and benefits of participation in our trial. None of the participants received any direct compensation for their participation. Homeowners were, however, given €11.45 for raw material costs to fix damaged mud brick roofs or coat them with shea butter as was the custom following the rainy season. Every participant is free to refuse or interrupt data collection at any point in time. A decision not to participate in this study will not bear any further consequences for the individual. We explained that participation in our current trial can potentially generate the benefit of a cooler house, which can lead to health benefits for participants as a result of their participation. There will be no special criteria for discontinuing or modifying the allocated interventions. We will, however, track reasons for attrition and report these in subsequent publications.

After study completion, we will provide relatable summaries of study results to acknowledge participants' contributions. Furthermore, we will provide our scientific findings to participants in an engagement workshop for community leaders, participants in the focus groups, and those involved in prognostication and co-production of interventions for further studies related to cool roofs. We anticipate that this feedback will be valuable to some participants because it will provide them with insights that can support future cool roofing initiatives. Regarding post-trial care, there is no anticipated harm and compensation for trial participation - study participants in the control arm will be offered a cool roof at the end of the trial, if we find positive effects.

No invasive procedures are to be borne by study participants other than those involved in routine diagnostic procedures, and no procedures other than those outlined here will be performed.

### Confidentiality

Complete confidentiality of data will apply, and the data collection will conform to requirements of the national and federal legislations on data protection. We are storing digital data on password-protected files. Data is available exclusively to the research team members for complete confidentiality. Third parties will receive anonymized data only for research purposes. Informed consent forms, laboratory books, and other participant-related documents will be safely stored during study conduct and, subsequently, at the Co-Principal Investigator’s office premises.

### Dissemination

Upon completion of the study, participant-related information on results will be provided to the families and exclusively by the clinical officer in charge or the Principal Investigator. Results will also be presented at international peer-reviewed journals, national and international conferences (ranging from academic conferences to United Nations Climate Change Conference (COP) meetings), our project website, and through media publications and interviews [54].

## Discussion and policy implications

Our cRCT described here tests whether a passive home cooling technology — cool roofs — reduces indoor ambient temperature and improves cardiovascular, metabolic, sleep, mental health, and general health outcomes in Nouna, Burkina Faso- a region highly vulnerable to increasing heat exposure from climate change. When applied to homes as described in this study, cool roofs may serve as a feasible and scalable intervention for adapting to and ameliorating the effects of extreme heat. The impact, in practice, depends on the uptake. Cool roofs are attractive as a passive, inexpensive technology that does not require behavior change and carries desirable co-benefits such as roof waterproofing and indoor noise reduction. Our trial is an interdisciplinary project bringing together elements of public health, medicine, occupational health, engineering, materials science, development, and public policy. We linked social science and epidemiology to develop the trial design; engineering and material science to understand building design and heat transfer; natural science and occupational health to define endpoints and understand heat effects on health and productivity; and development and public policy to engage decision-makers and donors.

The most important facet for intervention uptake is community buy-in, which stems from our participatory research model — where we are actively engaging and partnering with local stakeholders and community members. Through this partnership model, we are gaining an understanding of community adaptation needs to reduce adverse health effects of heat exposure. We designed all project activities to give Nouna community members a sense of agency, control, and empowerment. We trained and employed local painters to apply the cool roof coating successfully. We planned the logistics of coating procurement, storage, distribution, application, and maintenance (in case of damages) to ensure the coating application was finalized in a short window between COVID-19 field work restrictions ending and the commencement of the rainy season. Participants were compensated for materials to repair roof damages following the rainy season. We trained local interviewers for monthly home visits, and local field staff were also trained exclusively in retrieving smartwatch data. Two local entomological field teams were also responsible for collecting mosquito specimens. The intervention was introduced through focus group discussions, community leader interactions, and home visits, often using cool roof samples to showcase the technology. We created a 5-min video of our cool roof adaptation trial to engage a broader audience [55].

Adaptation is necessary to reduce the harm caused by climate change, but adaptative capabilities are distributed inequitably, and resource-constrained regions are often those least able to adapt [56, 57]. Despite the need to test and deploy adaptation interventions in Africa, current initiatives are insufficient. Complex field trials on climate change adaptation rarely prioritize the most vulnerable populations, such as those in Nouna, Burkina Faso. Yet, this is where trials are most needed [58]. Between 1991 and 2021, the average surface temperature over the African continent has risen 0.3 °C per decade faster than the global average, and the frequency of extremely hot days also has increased [59]. Projections indicate these trends will continue into the future — leading to adverse health effects [60]. Furthermore, there may be devastating effects on populations due to disrupted agriculture/food security, conflict, and displacement [61–63]. In response, more than 40 African nations have committed to more ambitious adaptation efforts in their national adaptation plans (NAPs), and the cool roofs project in Nouna demonstrates it is possible to conduct field trials in a region facing climate, security, and political challenges.

Strengthening local capacity and bolstering NAPs requires improving pathways for the transfer of low-carbon technology to regions like Nouna. As with potentially desirable low-carbon technologies, cool roof coatings are not currently manufactured locally in Burkina Faso. In our trial, we have successfully engaged with a European non-governmental organization — the European Cool Roof Council (ECRC) — that has the mandate to promote the use and expansion of low-carbon technology, such as cool roofs, in the European Union. Three ECRC member companies — Sika AG, Engineered Polymer Solutions B.V., and SOPREMA SAS — expressed interest in sponsoring cool roof coatings for our trial free of charge (as unrestricted educational grants). Our engagement is the first step in building awareness of the need for low-carbon technologies such as cool roofs in Africa, and it highlights the importance of developing public–private partnerships for embedding low-carbon technology solutions in local African environments.

In addition to involving local communities and industry, we must engage policymakers. In New Zealand, RCTs investigating the impact of installing insulation or non-polluting home heating on respiratory outcomes were instrumental in creating a government subsidy scheme for low-income households to receive insulation and heating grants [64–66]. These studies demonstrated the power of intervention research, providing robust evidence for governments to formulate effective policies. Considering the deficiency of local empirical trials on housing adaptation and health in climate-vulnerable African regions, our study will be integral in shaping policy development in this region [67]. To ensure the policy relevance of our research, we have engaged the Director General of the National Institute of Public Health in Burkina Faso to drive collective responses to observed impacts and provide resources to facilitate adaptation and manage access to resources [67]. Since establishing the trial, we have engaged government agencies in Burkina Faso, including the Ministry of Health and the Ministry of Aviation and Transport. We will further engage the Ministry of Environment, Green Economy, and Climate Change of Burkina Faso. Our trial has also been featured in the Global Heat Health Information Network (GHHIN) monthly digest and at the 2021 United Nations Climate Change Conference (COP26) event on climate change adaptation in Africa.

Sociodemographic structure, local economic forces, and community values are driving forces mediating adaptive capacity to climate change, especially in Burkina Faso [68, 69]. These factors highlight the need for simple interventions, such as cool roofs that improve adaptive capacity uniformly across populations. Though inexpensive technologies for climate change adaptation exist research on the effects of these interventions in resource-constrained regions of the globe is limited. Even less well-studied are the downstream improvements these interventions can have on health. Evidence demonstrating the effectiveness of adaptation interventions such as cool roofs is critical for policymakers with limited budgets who must rely on proven technologies for health improvements. Our trial bridges this gap and provides causal, policy-relevant empirical evidence for the potential inclusion of cool roofs in adaptation policy packages in low-resource settings vulnerable to climate change.

## Trial status

Recruitment started on February 1, 2021, and was completed on March 31, 2021. Data collection ended on June 30, 2023. This version refers to version 1.5 of the approved protocol (August 16, 2021).

### Supplementary Information

**Additional file 1. **SPIRIT Checklist. 

## Data Availability

The research team will have access to the data, and anonymized datasets will be made available to the public and other researchers via an open-access repository in accordance with the FAIR (Findable, Accessible, Interoperable, Reusable) principles.

## Abbreviations

AIC: Akaike information criterion
CITI: Collaborative institutional training initiative
COVID-19: Coronavirus disease of 2019
CO_2_: Carbon dioxide
COP: United Nations climate change conference
COP26: 2021 United Nations climate change conference
cRCT: Community-based stratified clustered randomized control trial
DGF: Deutsche Forschungsgemeinschaft (German Research Foundation)
ECRC: European cool roof council
GLMM: Generalized linear mixed models
GHHIN: Global heat health information network
GPS: Global positioning system
GPT-3: Generative pre-trained transformer 3
HIGH: Heidelberg institute of global health
HbA1C: Hemoglobin A1C
HDSS: Health and demographic surveillance system
LAM: Liquid applied membranes
LMICS: Lower- and middle-income countries
NCDs: Non-communicable diseases
PI: Principal investigator
SRI: Solar reflectance index
SQL: Structured query language
